# Beyond One-Size-Fits-All: Targeted Dietary Interventions for Pediatric ADHD by Clinical Phenotype

**DOI:** 10.1192/j.eurpsy.2026.10561

**Published:** 2026-07-31

**Authors:** K. Kelly, A. Guo, S. Vendidandi, C. N. Noureddine, A. Sterchele

**Affiliations:** 1 Icahn School of Medicine at Mount Sinai, New York; 2 Rutgers New Jersey Medical School, Newark, United States

## Abstract

**Introduction:**

Dietary factors have gained increasing attention for their potential role in the development and management of psychiatric disorders, including attention-deficit/hyperactivity disorder (ADHD). This review aims to assess whether tailoring nutritional interventions to specific patient phenotypes enhances therapeutic efficacy in pediatric ADHD and to evaluate the impact of nutrition and diet on ADHD management in youth.

**Objectives:**

Determine whether tailoring nutritional interventions to specific patient phenotypes will enhance therapeutic efficacy in pediatric ADHD treatment.

Evaluate how nutrition and diet can impact the management and treatment of ADHD in youth.

**Methods:**

A literature review was conducted using Google Scholar and the National Institutes of Health PubMed database. Search terms included cross sections between “ADHD,” “attention deficit hyperactivity disorder,” and “nutrition” or “diet”, with “youth,” “adolescents,” “children,” and “pediatric” in various permutations. Of 621 search results, 83 articles relevant to the topic were included.

**Results:**

The few-foods diet (FFD) has been shown to significantly reduce somatic symptoms in children with ADHD, including eczema, diarrhea, and sleep disturbances. Nutritional supplements, particularly omega-3 fatty acids, have demonstrated efficacy in improving inattention, hyperactivity, and impulsivity. Micronutrient supplementation with iron, zinc, and magnesium has been linked to improvements in inattention and emotional dysregulation, particularly in those with deficiencies. These effects may be mediated by changes in the gut microbiome, as supplementation alters microbiota composition and evenness, with specific bacterial shifts associated with treatment response. Beyond FFD and supplementation, adherence to overall dietary patterns, including the Mediterranean diet, has been correlated with reduced odds of ADHD in children.

**Conclusions:**

Nutritional interventions show promise in pediatric ADHD, with emerging evidence that clinical phenotypes may guide treatment selection. Targeted supplementation may be more effective in children with micronutrient deficiencies or low omega-3 levels. The FFD may provide greater benefit in children with atopic comorbidities, while Mediterranean diets show modest effects broadly across ADHD populations. Further randomized controlled trials specifically designed to validate differential responses across ADHD subgroups are warranted to improve the precision-based dietary guidelines.

**Disclosure of Interest:**

None Declared

